# Case report: A novel *FARS2* deletion and a missense variant in a child with complicated, rapidly progressive spastic paraplegia

**DOI:** 10.3389/fgene.2023.1130687

**Published:** 2023-04-19

**Authors:** Elena Panzeri, Andrea Citterio, Andrea Martinuzzi, Vera Ancona, Eleonora Martini, Maria Teresa Bassi

**Affiliations:** ^1^ Laboratory of Molecular Biology, Scientific Institute IRCCS E. Medea, Bosisio Parini, Italy; ^2^ Department of Neurorehabilitation, Scientific Institute IRCCS E. Medea, Conegliano, Italy

**Keywords:** complicated spastic paraplegia, *FARS2*, deletion, SPG77, severe

## Abstract

Defects in *FARS2* are associated with either epileptic phenotypes or a spastic paraplegia subtype known as SPG77. Here, we describe an 8-year-old patient with severe and complicated spastic paraplegia, carrying a missense variant (p.Pro361Leu) and a novel intragenic deletion in *FARS2.* Of note, the disease is unexpectedly progressing rapidly and in a biphasic way differently from the previously reported cases. Our study provides the first detailed molecular characterization of a *FARS2* deletion and its underlying molecular mechanism, and demonstrates the need for combining different tools to improve the diagnostic rate.

## Introduction

**FIGURE 1:**
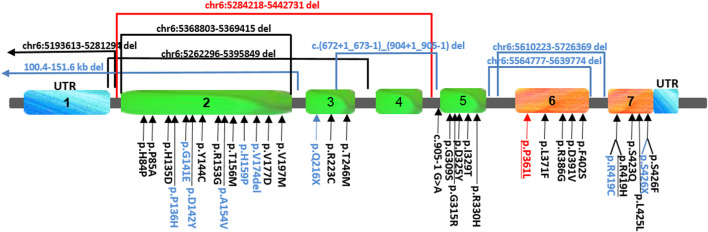
Schematic view and location of the *FARS2* variants reported so far. The variants found in our patient are indicated in red, those found either in epileptic or spastic patients are underlined, and those found only in epileptic patients are indicated in black, while the variants associated only with spastic paraplegia are indicated in blue. To date, 41 pathogenic *FARS2* variants have been found to be spread throughout the gene, including missense, non-sense, and splice-site variants, and deletions.

The human *FARS2* gene encodes mitochondrial phenylalanyl-tRNA synthetase, which contributes to the accuracy of the translation of mitochondrial proteins, avoiding ATP deficiency in the cells (Roy et al., 2005). To date, 41 pathogenic *FARS2* variants, inherited in an autosomal recessive pattern, have been reported (Figure 1). The variants are related to three main phenotypic manifestations: infantile-onset epileptic mitochondrial encephalopathy, later-onset spastic paraplegia (SPG77), and juvenile-onset refractory epilepsy. Usually, the epileptic groups have a poorer prognosis, while SPG77 is associated with a less severe disease and prolonged survival (Shamseldin et al., 2012; Yang et al., 2016; Hotait et al., 2020). Here, we describe a patient affected by a complicated and rapidly progressive form of hereditary spastic paraplegia (HSP) with upper limb involvement, bilateral single palmar creases, clinodactyly, mild intellectual disability, and dysmorphic features, carrying compound heterozygous variants in *FARS2*, the known missense variant p.Pro361Leu, and a novel intragenic deletion involving the coding exons 2, 3, and 4. Herein, we provide the first detailed molecular characterization of the intragenic deletion, identifying the molecular mechanism underlying the genomic rearrangement.

## Materials and methods

### Case presentation

**FIGURE 2:**
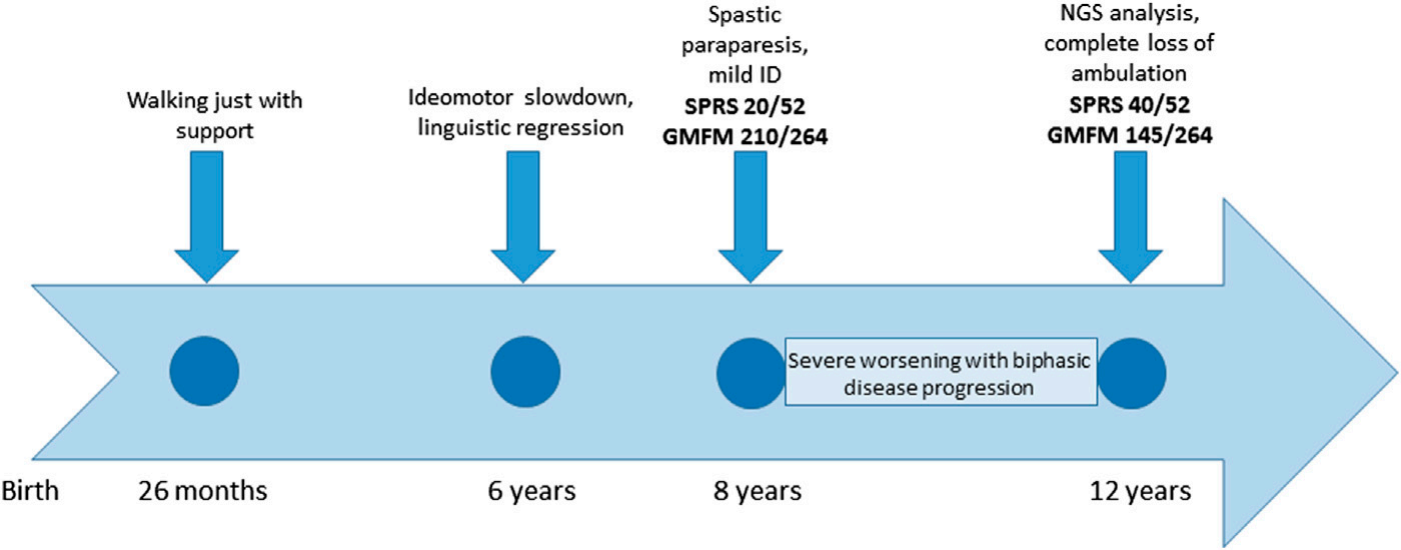
Timeline with relevant episodes in the case report presented.

The patient was referred to our clinic at the age of 8 years for cerebral palsy and developmental delay. The patient has a healthy brother and sister aged 18 and 15 years, respectively. The child was born at term, the pregnancy was uneventful, and the delivery and postnatal period had been uncomplicated. The child began walking with support at 26 months of age. The subject was admitted to an outpatient rehabilitation program since 2 years of age. At 6 years of age, the patient showed transient signs of ideomotor slowdown and linguistic regression, associated with nocturnal awakenings that spontaneously resolved over 2 weeks. The brain magnetic resonance imaging (MRI) and electroencephalogram (EEG) results were negative. The patient was diagnosed with a complicated and rapidly progressive form of HSP with upper limb involvement, dyspraxia, irregular action tremor, pronounced pyramidal signs in the lower limbs (deep tendon hyperreflexia, ankle clonus, and Babinski sign), bradykinesia, dorsal kyphoscoliosis, pronated valgus feet, and postural imbalance. Gait analysis at 8 years of age detected spasticity of hamstring/gastrocnemius muscles and a weakness of the dorsal flexors of the feet. In spite of this, autonomous gait was still possible; the spastic paraplegia rating scale (SPRS) score was 20/52, and the gross motor function measure (GMFM) score was 210/264. The child also presented bilateral single palmar creases on their hands, clinodactyly, mild intellectual disability, and dysmorphic features (macrocephaly, micrognathia, ogival palate, and macrodontia with severe caries). The subject’s social skills were fairly expressed, but verbal understanding was limited; speech was markedly dysarthric and not always intelligible. Creatine kinase, thyroid hormones, and sensory and motor nerve conduction velocities (NCVs) of the lower limbs were normal. Needle electromyography did not show any sign of active denervation. The child had exophoria and myopic astigmatism, but the fundus oculi results were negative. There was no sensory deficit. At the last clinical evaluation at the age of 12 years, a significant progression of the disability was evident, with a complete loss of ambulation. The SPRS score was 40/52, and the GMFM score was 145/264 (timeline in Figure 2). A unique aspect of this case is the modality of disease progression, apparently in a biphasic pattern, with decreasing development in the first 8 years of life, when an autonomous gait was still possible, followed by a very rapid worsening of the motor function with a complete loss of ambulation by the age of 12 years.

### Genetic analysis

Genomic DNA (gDNA) was extracted from the patient’s peripheral blood and screened using a targeted next-generation sequencing (NGS) approach with a custom gene panel from Agilent Technologies (Santa Clara, CA, United States), including 231 genes related to HSP, ataxias, and neuropathies (Supplementary Material). The targeted regions were run on a NextSeq platform (Illumina, San Diego, CA, United States), and the variants located in the coding regions, including the splice site, that exhibited a MAF <1% or were not present in variant databases were then analyzed with different tools to predict a possible pathogenicity. Sanger sequencing was used to confirm the variant in the patient and to verify the segregation in the patient’s family. The data were then further analyzed with ExomeDepth, a powerful bioinformatic tool, which is able to detect even small copy number variants (CNVs) using a robust beta-binomial model (Plagnol et al., 2012). Every CNV called with a Bayes factor (BF) higher than 20 was tested by qPCR. In view of the complicated features of the patient (developmental delay, dysmorphic features, intellectual disability, and skeletal abnormalities) and of the early onset of the disease, we also performed array CGH and whole-exome sequencing (WES) to exclude a possible coexistence of other genetic abnormalities (see Supplementary Material for a detailed description of the materials and methods used in this study).

### Real-time PCR

To validate the deletion found with ExomeDepth, we performed a quantitative PCR (qPCR) on three regions downstream of exon 1 and three regions downstream of exon 4 within the putative deleted region. The primer pairs for qPCR analysis were selected within the non-repeated portions of the chromosome by Primer Express software (Applied Biosystems, Foster City, CA, United States) (See Supplementary Material for a detailed description).

### Breakpoint characterization

A long-range PCR was performed with Herculase II fusion DNA polymerase (Agilent Technologies, Santa Clara, CA, United States), following the protocol for a fragment larger than 10 kb (Supplementary Material). Amplification was then tested by electrophoresis with 0.8% agarose gel, and the smaller size DNA fragment, found only in the proband, was sequenced with a BigDye Terminator Cycle Sequencing Kit (Applied Biosystems, Waltham, MA, United States) and run on an ABI 3500xL Genetic Analyzer. *In silico* analysis carried out with the RepeatMasker program (http://www.repeatmasker.org) and CENSOR utility (http://ebi.acuk/Tools/censor) were used to find possible elements of homology in the breakpoint regions. BLAST2 software (http://www.ncbi.nml.nih.gov/BLAST/) was used to align sequences close to the two breakpoints.

### RNA extraction and cDNA sequencing

RNA was obtained from patient’s skin fibroblasts and from the PAXgene blood samples from their parents and extracted using either the Direct-zol RNA MiniPrep Kit (Zymo Research, Irvine, CA, United States) or the QIAGEN PAXgene Blood RNA Kit (PreAnalytiX, QIAGEN, Hombrechtikon, CH). A volume of 1 μg of RNA/sample was reverse-transcribed into cDNA using the SuperScript First-Strand Synthesis System for the reverse transcription (RT)-PCR kit (Thermo Fisher Scientific, Waltham, MA) and used for the PCR (Supplementary Material).

## Results

**FIGURE 3:**
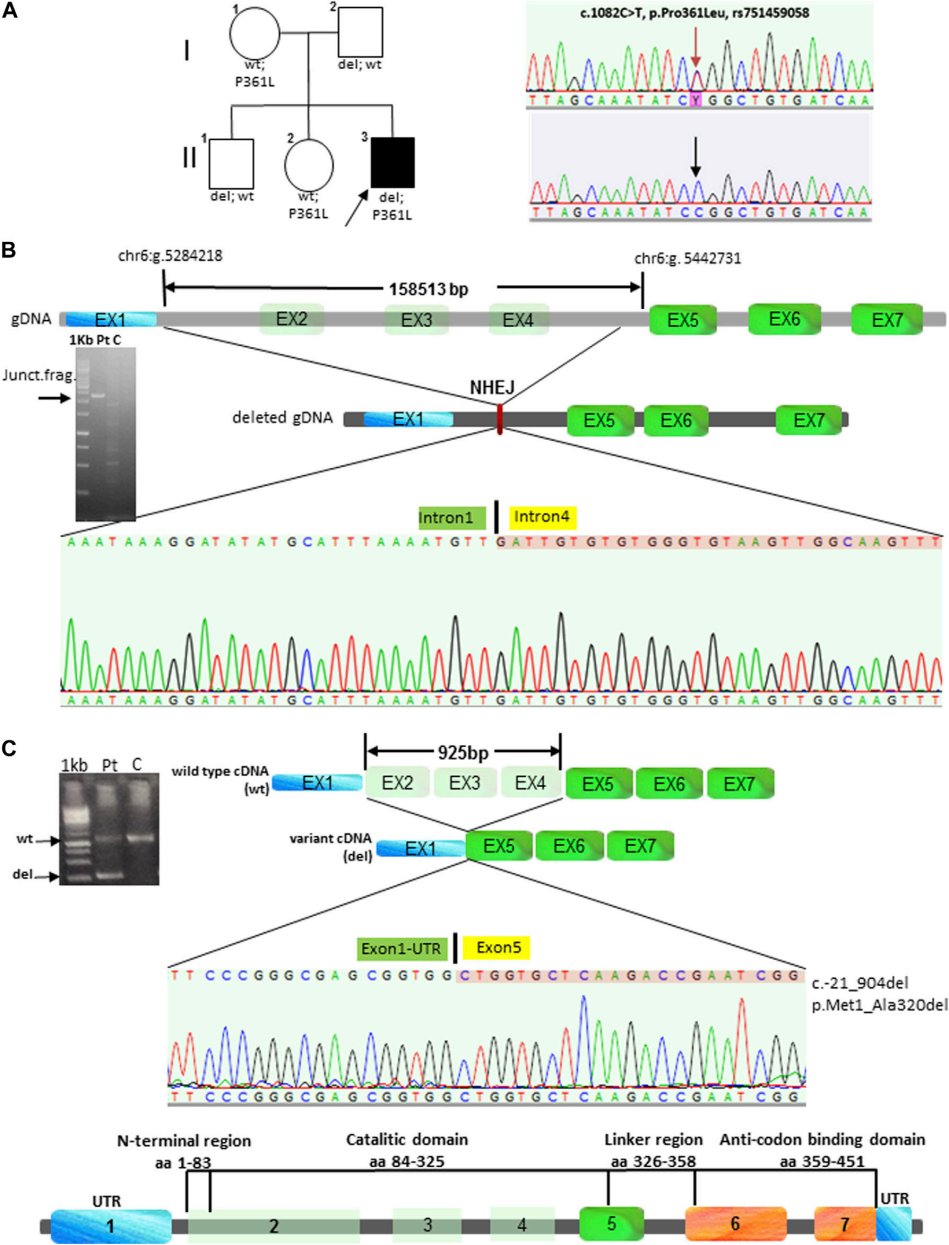
**(A)** On the left, the family pedigree with the proband is indicated by an arrow and a black square. The non-affected individuals are indicated by unfilled symbols. On the right, electropherogram of the *FARS2* p.Pro361Leu maternal variant found in the patient was compared to the wild-type sequence (wt). **(B)** Schematic view of *FARS2* at the genomic level with an indication of breakpoint locations, deleted exons in light green, and hypotheses of the underlying genomic event (non-homologous end joining, NHEJ). gDNA electrophoresis of the junction fragment and the schematic view of the gDNA resulting from the deletion are shown here. In the following figure, we see electropherogram of the sequence generated from the deletion, which spans from intron 1 to 4 of *FARS2*. **(C)** On the left, we observe the cDNA electrophoresis of the wild-type sequence and the fragment generated by the paternal intragenic deletion (del). On the right, we observe the schematic view of *FARS2* at the cDNA level with the indication of the deleted exons in light green and representation of the cDNA resulting from the deletion. In the following figure, we see electropherograms of the 925 nucleotide deletion that lead to the loss of exons 2–3–4, including the canonical starting codon in exon 2. An ATG, located 56 nucleotides downstream of the breakpoint, might generate a putative protein with 131 residues instead of the canonical 451. This putative protein product (if any) is likely inactive because it lacks the catalytic functional domain, while it retains the FARS2 linker region and the anticodon-binding domain as shown here.

A single heterozygous pathogenic variant was found in *FARS2*, c.1082C>T (p.Pro361Leu) (Figure 3A); the subsequent search for CNVs using the ExomeDepth tool led to the identification of an intragenic deletion spanning the region from intron 1 to 4 of *FARS2*. Amplification, subcloning, and sequencing of the junction fragment at the breakpoint revealed a deletion spanning 158,513 bp at the genomic level (g.5284218-5442731del). The absence of any repeats or homology regions suggested that a non-homologous recombination event had likely mediated the rearrangement (Figure 3B). At the cDNA level, the deletion involved 925 nucleotides (c.21_904del), eliminating the first three coding exons of the gene (exon 2 containing the ATG, and exons 3 and 4), corresponding to the first 319 amino acid residues of FARS2 protein. An in-frame ATG, 56 nucleotides downstream of the breakpoint, is still maintained, and a putative polypeptide containing the last 131 amino acids of FARS2 might still have been translated. This putative protein product (if any) is likely inactive because it lacks the catalytic functional domain (p.Met1_Ala320del), while it retains the FARS2 linker region and the anticodon-binding domain (Figure 3C). Segregation analysis showed that the missense variant was maternally inherited and transmitted to the proband’s unaffected sister, while the deletion was paternally inherited and segregated in the proband’s unaffected brother (Figure 3A). We confirmed these data by performing WES and array CGH on the trio. No additional pathogenic variants or CNVs were identified.

## Discussion

Several *FARS2* patients have been described with clinical phenotypes ranging from different types of epilepsy to pure and complicated forms of HSP SPG77. To date, 41 pathogenic *FARS2* variants were found, including missense, nonsense, splice-site variants, and deletions. Among all the variants, seven are deletions and four of them are associated with SPG77 (Vernon et al., 2015; Forman et al., 2019; Jou et al., 2019; Meszarosova et al., 2020) (Figure 1). Although the deletions were not molecularly characterized with breakpoint subcloning and sequencing, in one case (Meszarosova et al., 2020), the analysis of genomic sequences surrounding the deletion had revealed the presence of highly homologous ALU repeats in *FARS2* intron 2 and in *LYRM4* intron 1, likely mediating the rearrangement. Here, we describe a child affected by a complicated form of SPG77, carrying compound heterozygous variants in *FARS2*, the known p.Pro361Leu pathogenic variant, and a novel intragenic deletion of 158513 bp, involving the first three coding exons of the gene, including the start codon. Unlike the case reported by Meszarosova and colleagues, the breakpoint sequence analysis in our patient revealed that the rearrangement was not mediated by any homologous region, thereby indicating a non-homologous end joining (NHEJ) recombination event had likely occurred. The NHEJ pathway is well known to be the election pathway for repairing double-strand breaks because their misrepair can cause severe changes, such as deletions or chromosomal translocations (Shibata, 2017). Based on the putative effects of *FARS2* variants, we hypothesize a dramatic decrease of the FARS2 protein function in our patient. Indeed, the maternal p.Pro361Leu allele may likely retain a residual activity since this variant was already reported as affecting the stability of the anticodon-binding domain, thereby leading to a decreased protein interaction with tRNAPhe (Vantroys et al., 2017), while the paternally deleted allele may generate a likely putative inactive protein (if any) without the functional catalytic domain. This genotype is associated with clinical features partially overlapping those of a family reported by Vernon and colleagues (Table 1), with an early-onset severe spastic paraplegia, global developmental delay, and several dysmorphisms. In addition, our patient showed mild intellectual disability and additional dysmorphic features, such as bilateral single palmar creases on the hands, and clinodactily that were not observed in patients reported by Vernon and colleagues. Furthermore, a unique aspect of this case is the disease that seems to progress in a biphasic pattern, with decreasing development in the first 8 years of life, when an autonomous gait was still possible, followed by a very rapid worsening of the motor function with a complete loss of ambulation by the age of 12 years. Considering the clinical features of all SPG77 patients described so far, including the novel variant reported here, genotype–phenotype correlations are difficult to be established, given that the variants, such as p.Arg419His, are associated with both HSP and epileptic encephalopathy (Jou et al., 2019; Barcia et al., 2021). Analogously, even when considering only the HSP-related variants, there is no clear correlation between the genetic defect and disease severity, with the p.Pro361Leu variant (also carried by the patient described here) found in both pure and complicated forms (Table 1). Furthermore, neither the presence of a deletion nor its size (large ones or single amino acid deletions) could be correlated with a specific phenotype. Indeed, the loss of a single residue (p.Val174del) is associated with a complicated HSP form with a very early onset and severe presentation (Vantroys et al., 2017), while a deletion of about 151.6 kb was found in a patient with a pure form of HSP (Meszarosova et al., 2020). Thus, the phenotypic differences observed may be due to different combinations of *FARS2* variants, as previously postulated (Barcia et al., 2021). Overall, the clinical and genetic data presented here further confirm the lack of any genotype–phenotype correlations in *FARS2* patients. This represents a major drawback in genetic counseling and clinical prognoses in these patients. Therefore, a better understanding of the molecular mechanism(s) of *FARS2* variants should allow a better risk assessment for patients and their families. In addition, this may also lead to the development of novel therapies. To this aim, the *Drosophila dFARS2* mutants, mimicking many disease features, might represent a useful tool to better define the pathogenic mechanisms underlying the variable degree of *FARS2*-related disease severity (Fan et al., 2021).

**TABLE 1:** Overview of the *FARS2* variations related to SPG77 to date.

Reference	Onset	HSP	Other clinical findings	*FARS2* variant
Yang et al. (2016)	2 y	Pure	Progressive lower limb spasticity, pyramidal weakness with hyperreflexia, extensor plantar responses, and scissor gait	p.Asp142Tyr/p.Asp142Tyr
	1 y			
	5 y			
	3 y			
Vernon et al. (2015)	2 m	Complicated	Seizures, DD, dysarthria, strabismus, macrodontia, retrognathia, truncal hypotonia, intention tremor, bilateral equinovarus deformity, and hip dysplasia only in the sister; scoliosis, ptosis, and multiple scattered nevi only in the brother	chr6:5610223-5726369del/p.Arg419Cys
	6 w			
Vantroys et al. (2017)	6 m	Complicated	Wheelchair-bound (8 years), lower extremity hyperreflexia, DD, poor head control, progressive kyphoscoliosis, bilateral cryptorchidism, and seizures	p.Ala154Val/p.Pro361Leu
	10 m	Complicated	Bilateral talipes equinovarus, poor feeding, DD, wheelchair-bound (6 years), severe insomnia, bradykinesia, tremor, dystonia, and dysarthria (15 years)	p.Val174del/p.Pro361Leu
Sahai et al. (2018)	2.5 y	Pure	Spastic gait with toe walking, lower limb spasticity, and brisk deep tendon reflexes	p.Pro136His/p.Arg216Ter
Almannai et al. (2018)	2 y	Complicated	DD and abnormal gait	p.His159Pro/p.Arg419Cys
	1 y			
Forman et al. (2019)	13 y	Complicated	DD, weakness of lower limbs, brisk deep tendon reflexes, extensor plantar responses, and clonus; the brother was wheelchair-bound, with tremors and dysphonia (12 years)	p.Gly141Glu/chr6:5564777-5639774del
	7 y			
Jou et al., 2019	7 y	Complicated	Encephalopathy	chr6:5404834-5431406del/p.Arg419His
Meszarosova et al. (2020)	5 y	Pure	Gait impairment, bilateral hyperreflexia, Babinski sign, clonus, pes cavus, lower limb mild muscle atrophy, and mildly increased muscle tonus in the legs	chr6:5244167-5395818(?)/p.Pro361Leu
Barcia et al. (2021)	8 y	Complicated	Axial hypotonia, DD, dystonic movements, wheelchair-bound, and dysarthria	p.Arg419His/p.Ser426Ter
Kartvelishvili et al. (2017)	17 y	Complicated	DD, encephalopathy, migraine, pyramidal signs, dystonia, and gonadal failure	p.His159Pro/p.Arg419Cys
This study	26 m	Complicated	Deep tendon hyperreflexia, clonus, Babinski sign, bradykinesia, gait imbalance, DD, mild ID, macrocephaly, dorsal kyphoscoliosis, pronated valgus feet, simian hands, micrognathia, ogival palate, macrodontia with severe caries, clinodactyly, multiple nevi, dysarthria, dyspraxia, tremor, dysgraphia, loss of ambulation, and fast and biphasic progression	p.Met1_Ala320del/p.Pro361Leu

y, years; m, months; w, weeks; DD, developmental delay; ID, intellectual disability.

## Conclusion

Our study widens the phenotypic spectrum and heterogeneity associated with *FARS2* variants. Furthermore, we report the first molecular characterization of a *FARS2* deletion and stress on the importance of combining different tools (i.e., sequencing and deletion/duplication studies) for variant screening in HSP patients, especially when the gene panels are inconclusive.

## Data Availability

The original contributions presented in the study are included in the article/Supplementary Material, further inquiries can be directed to the corresponding author.
