# Supplementary material for: Case report: A novel FARS2 deletion and a missense variant in a child with complicated, rapidly progressive spastic paraplegia
Source: Front Genet. 2023 Apr 19;14:1130687. doi: 10.3389/fgene.2023.1130687 (PMC10154595; doi:10.3389/fgene.2023.1130687)
Supplement: Supplementary file 1 [file DataSheet1.docx]

**Supplementary Material**

**Clinical data**

At the age of 8 years the proband was referred to our attention for cerebral palsy and developmental delay. The child had a brother and a sister aged 18 and 15 respectively. No anamnestic data of pathology was found in the mother and older siblings. The father had exophoria, hypercholesterolemia and multiple coronary stents implanted at 42 years of age. The father's brother had suffered from “absence” epilepsy, mild intellectual disability, claw hands, severe fine motor dyspraxia, and dysgraphia. He died in adulthood owing to severe early-onset heart disease as reported in the proband’s paternal grandfather and great-grandfather.

The patient was born at term to a third uneventful pregnancy. Delivery and postnatal period had been uncomplicated. He had no sleep, feeding or growth disorder. Walking had been possible just with support since 26 months of age. He had mild intellectual disability and he was not independent in learning and daily activities.

At 6 years of age, the child had transient signs of ideomotor slowdown and linguistic regression. These clinical manifestations were associated with nocturnal awakenings and spontaneously resolved over two weeks. Brain MRI and electroencephalogram then performed were negative.

At our clinical examination several nevi were noticed. Head circumference was at 75th percentile. The patient had micrognathia, ogival palate, macrodontia with severe caries, dorsal kyphoscoliosis, pronated valgus feet, bilateral single palmar creases in hands and clinodactyly (V finger of the hands, II and III finger of the feet). Social skills were fairly expressed, but verbal understanding was limited. Speech was definitely dysarthric and not always intelligible. The child had exophoria and myopic astigmatism; fundus oculi was negative. Ocular, bucco-facial, gross and fine motor dyspraxia were observed, with irregular action tremor and dysgraphia. Overall, a spastic paraparesis and pronounced pyramidal signs of lower limbs (deep tendon hyperreflexia, ankle clonus, Babinski sign) were the main neurological traits of the phenotype. Bradykinesia as well as postural and gait imbalance were associated. There was no sensory deficit. Creatine kinase, thyroid hormones, sensory and motor nerve conduction velocities (NCV) of lower limbs were normal. Gait analysis performed when the child was 8 years old detected spasticity of hamstring/gastrocnemius muscles and hyposthenia of dorsal flexors of the feet. In spite of this, autonomous gait was still possible, spastic paraplegia rating scale (SPRS) score was 20/52 and the gross motor function measure (GMFM) score was 210/264. The needle electromyography did not show any sign of active denervation.

At the last clinical evaluation at age 12 years significant progression of disability was evident, with complete loss of deambulation. SPRS score was 40/52 and GMFM score was 145/264.

**NGS sequencing**

DNA was extracted from patient’s peripheral blood and was screened using a targeted next generation sequencing (NGS) approach with a gene panel including 231 genes: all known causative genes for hereditary spastic paraplegias (HSP), the known genes for recessive ataxias and for spinocerebellar ataxias (excluding those forms related to repeat expansion), the most frequently mutated genes in neuropathies and the known genes for familial amyotrophic lateral sclerosis (ALS). The gene list is reported below. The targeted regions were designed to include coding exons with intronic 20 bp flanking sites. The sequencing libraries were prepared from genomic DNA using SureSelect enrichment system (Agilent Technologies, Santa Clara, CA, USA) and run on NextSeq platform according to the manufacturer's instructions (Illumina, San Diego, CA, USA). The sequenced reads were then aligned to reference target regions and variant were called with BWA enrichment application (which include also GATK for variant calling) available on BaseSpace Sequence HUB (Illumina, San Diego, CA, USA). ANNOVAR was used for annotation against the RefSeq and the Single Nucleotide Polymorphism databases. The filtering strategy we applied led us to select only variants located in the coding regions, including the splice site, that exhibited a MAF <1% or were not present in variant databases including those of the 1,000 Genomes Project and Genome Aggregation database gnomAD. Synonymous variants were excluded.

Selected variants were then analyzed with different tools to predict possible pathogenicity such as DANN, DEOGEN2, EIGEN, FATHMM-MKL, M-CAP, MVP, MutationAssessor, MutationTaster, PrimateAI, REVEL, Polyphen-2 and SIFT.

Variants were classified according to the American College of Human Society (ACMG) guidelines and segregation was verified by Sanger sequencing in the family members.

The deletion was identified using ExomeDepth, a powerful bioinformatic tool (coded within an R-package) which maximize the statistical power to detect even small CNVs using a robust beta-binomial model. The tool is realized to identify CNVs in exome samples, but could be successfully used also with gene panels NGS data, combing several gene panels data (16 control males and 16 control females), resulted negative to aCGH analysis, in order to construct a broad reference set to base the CNV inference on and using default parameters to create count data from BAM file and for CNV calling. Based on manufacturer’s instructions we set the cutoff of the resulted Bayes factor (BF) on 20 (which indicates the confidence range for the presence of a CNV). ^9^ Every CNVs called with a BF higher than 20 had been tested by qPCR.

In view of the complicated features of the patient (developmental delay, dysmorphic features, intellectual disability and skeletal abnormalities) and of the early onset of the disease, we performed array-CGH and whole exome sequencing (WES) to exclude a possible coexistence of other genetic abnormalities.

For whole exome sequencing, DNA fragmented libraries were amplified using the SureSelect Human All Exon V8 (Agilent Technologies), sequenced on a NextSeq platform (Illumina) and analysis was performed as already described for HSP panel.

Array-CGH was performed using Agilent Human Genome CGH Microarray Kit 4 × 180 k with an overall median probe space of 13 Kb.

**Gene list (231) of the targeted NGS panel**

*AARS, ABCD1, ADAR, ADD3, AFG3L2, ALDH18A1, ALS2, AMPD2, ANO10, AP4B1, AP4E1, AP4M1, AP4S1, AP5Z1, APTX, ARHGEF10, ARID1A, ARID1B, ARL6IP1, ARSI, ASAH1, ATL1, ATL3, ATM, ATN1, ATP13A2, ATP1A2, ATXN10, B4GALNT1, BICD2, BSCL2, C10ORF2, C12ORF65, C19ORF12, CABC1, CACNA1A, CACNA1G, CACNB4, CAPN1, CASK, CAV1, CCDC88C, CHCHD10, CHMP1A, CHP1, COA7, COASY, CP, CPT1C, CYP27A1, CYP2U1, CYP7B1, DARS, DARS2, DCAF17, DCTN1, DDHD1, DDHD2, DGAT2, DNM2, DNMT1, DSTYK, DYNC1H1, EEF2, EGR2, ELOVL4, ELOVL5, ENTPD1, EP300, EPT1, ERLIN1, ERLIN2, EXOSC3, FA2H, FARS2, FAT1, FAT2, FGD4, FGF14, FIG4, FLRT1, FMR1, FTL, FXN, GAN, GARS, GBA2, GBE1, GDAP1, GDAP2, GJB1, GJC2, GRID2, GRM1, GRN, GSN, HEXA, HEXB, HSPB1, HSPB3, HSPB8, HSPD1, IBA57, IFRD1, IGHMBP2, ITPR1, KANK1, KCNA1, KCNC3, KCND3, KIAA0196, KIAA0226, KIF1A, KIF1B, KIF1C, KIF26B, KIF5A, L1CAM, LITAF, LMNA, MAG, MARS, MARS2, MFN2, MME, MORC2, MPZ, MRE11A, MSTO1, MTHFR, MTPAP, MTTP, NARS2, NEFL, NEK1, NFASC, NIPA1, NOP56, NPC1, NT5C2, OPA1, OPTN, PANK2, PDYN, PEX10, PEX7, PGAP1, PHF21A, PHYH, PLA2G6, PLD3, PLP1, PLXNA2, PMM2, PMP22, PNPLA6, POLG, POLR3A, POLR3B, PRKCG, PRRT2, PYGM, RAB3GAP2, RAB7A, RARS2, REEP1, REEP2, RNF170, RTN2, SACS, SARS2, SCP2, SCYL1, SEPSECS, SETX, SH3TC2, SIGMAR1, SIL1, SLC1A3, SLC2A1, SLC33A1, SLC9A6, SOD1, SPAST, SPG11, SPG20, SPG21, SPG7, SPTAN1, SPTBN2, STUB1, SYNE1, SYNE2, TACO1, TBCD, TBCE, TBK1, TBP, TDP1, TECPR2, TFG, TGM6, TK2, TMEM240, TRIM2, TRMT5, TRPC3, TRPV4, TSEN2, TSEN34, TSEN54, TTBK2, TTPA, TUBA4A, TUBB2A, UBA5, UBR4, USP8, VAMP1, VAPB, VCP, VPS37A, VRK1, WDR45B, WDR48, XRCC1, XRCC4, YARS, ZFR, ZFYVE26, ZFYVE27*.

**Real-time PCR**

To validate the deletion found with ExomeDepth we performed a quantitative PCR (qPCR) on three regions downstream exon 1 and three regions downstream exon 4 within the putative deleted region. Primer pairs for qPCR analysis were selected within non-repeated portions of the chromosome using Primer Express software (Applied Biosystems, Foster City, CA, USA).

Primer downstream exon 1 of *FARS2*:

Forward-1 GCTTTGGTTTTCTGCAATAAGGA

Reverse-1 CCAGCACTCGTGGCAACA

Forward-2 CGAGTTTTGATCAGCTGGAGTGT

Reverse-2 GGGCCCAAGCCCCTTT

Forward-3 CCATGTGCCTGGCTCTAAGAA

Reverse-3 GCCCCCTTTGCTGTCACTT

Primer downstream exon 4 of *FARS2*:

Forward-4 GGCGCCACCTGACTGAGA

Reverse-4 TGCCTATAGATATGCGTAGAATTCAATC

Forward-5 CCCACCCTCGGTGACAGTT

Reverse-5 CCTAGTCAAAGAAGCACATCCTAGAGT

Forward-6 CGAAGCCGTGCCTGATTTA

Reverse-6 CACAGACCCCACCTGATGTCT

A control amplicon was selected with the same parameters in the *MAPK1* gene on 22q11.2; size (approximately 60 nt) and Tm (58°C) were the same for all amplicons. Amplification and detection were performed on a ABI QuantStudio3 Real Time PCR Instrument (Applied Biosystems, Waltham, MA, USA) using SYBR Green PCR Master Mix (Applied Biosystems, Waltham, MA, USA): thermal cycling conditions were 50°C for 2 min and 95°C for 10 min, followed by 40 cycles at 95°C for 15 sec and 60°C for 1 min. All samples (the tested sample, a male control and a female control) were amplified in duplicate. Validation experiments demonstrated that amplification efficiencies of the control and all target amplicons were approximately equal; accordingly, relative quantification of the amount of DNA was obtained using the ΔΔ threshold value (CT) method (see Table S1 for data).

To verify *FARS2* deletion segregation in the family, we performed qPCR used a set of primers located in the *MAPK1* gene as a control and with the following primers (see Table S2 for data):

Forward-2 CGAGTTTTGATCAGCTGGAGTGT

Reverse-2 GGGCCCAAGCCCCTTT

Table S1: raw rata of the qPCR performed to confirm the deletion region

| PRIMERS | SAMPLES | Gene expression |
| --- | --- | --- |
| ***FARS2* F1/R1** | Ctrl-M | 1 |
|  | Ctrl-F | 0,931172 |
|  | Patient | 1,159649 |
| ***FARS2* F2/R2** | Ctrl-M | 1 |
|  | Ctrl-F | 0,974018147 |
|  | Patient | 0,92113124 |
| ***FARS2* F3/R3** | Ctrl-M | 1 |
|  | Ctrl-F | 1,017341327 |
|  | Patient | 0,527579222 |
| ***FARS2* F4/R4** | Ctrl-M | 1 |
|  | Ctrl-F | 1,016005275 |
|  | Patient | 0,517737018 |
| ***FARS2* F5/R5** | Ctrl-M | 1 |
|  | Ctrl-F | 1,06626369 |
|  | Patient | 0,443816936 |
| ***FARS2* F6/R6** | Ctrl-M | 1 |
|  | Ctrl-F | 0,9614538 |
|  | Patient | 1,639065805 |

Table S2: raw rata of the qPCR performed to confirm the segregation of the deletion in the family members

| PRIMERS | SAMPLES | Gene expression |
| --- | --- | --- |
| ***FARS2* F2/R2** | Ctrl-M | 1 |
|  | Ctrl-F | 0,995809 |
|  | Patient | 0,558086 |
|  | Mother | 1,089507 |
|  | Father | 0,5053 |
|  | Brother | 0,528591 |
|  | Sister | 0,994315 |

**Breakpoint characterization**

A long-range PCR was performed with Herculase II Fusion DNA Polymerase following the PCR protocol for fragment larger than 10kb provided by the manufacturer (Agilent Technologies, Santa Clara, CA, USA) and using the cycling conditions for genomic DNA targets larger than 10kb. The primers used for amplifying fragments were:

Primer1 (sense): CGGAGTTGAGGGTTAGTGGT

Primer2 (antisense): ACTCACAAACTGTGCCAACA.

The amplification was then tested with electrophoresis on 0.8% agarose gel (Fig 2B) and the smaller size DNA fragment, present only in the proband DNA carrying the deletion, was sequenced with Big Dye Terminator Cycle Sequencing kit (Applied Biosystems, Waltham, MA, USA) and run on an ABI 3500xL Genetic Analyzer (Applied Biosystems, Waltham, MA, USA) using these primers:

Primer1 (sense): TCAGCTCTTACCTGGTCTTAG

Primer2 (antisense): GAGGGTAAATGCTTGAAAGTC.

The sequences were then aligned to the reference genome of *FARS2* with DNA Baser (Heracle BioSoft SRL 2020, RO) allowing us to identify the two breakpoints placed downstream exon 1 and exon 4. *In silico* analysis carried out with the RepeatMasker program (http://www.repeatmasker.org) and CENSOR utility (http://ebi.acuk/Tools/censor) were used to find possible elements of homology in the breakpoint regions. The BLAST2 software (http://www.ncbi.nml.nih.gov/BLAST/) was used to align sequences close to the two breakpoints.

**RNA extraction and cDNA sequencing**

RNA was obtained from patient’s skin fibroblasts and from PAXgene RNA tube blood samples from his parents. RNA was extracted using either Direct-Zol RNA MiniPrep Kit (Zymo Research, Irvine, CA, USA) or Qiagen PAXgene blood RNA Kit (PreAnalytix, Qiagen, Hombrechtikon, CH) following the manufacturers’ instruction.

RNA quality was assessed by Nanodrop 1000 (ThermoFisher Scientific, Waltham, MA) following the manufacturer’s instruction.

1 µg RNA/sample was reverse-transcribed into cDNA using the Superscript First Strand Synthesis System for reverse transcription (RT)-PCR kit (ThermoFisher Scientific, Waltham, MA) and random hexamers, then it was diluted 1:1 with TE for PCR.

PCR was performed using the primers *FARS2*-cDNA-Forward1: CGGTGCTGGGAGGGAGAT and *FARS2*-cDNA-Reverse1: GAGGCTGAAACTTCACCTTC and the products were then tested with electrophoresis on 1% agarose gel (Fig 2).
